# Pulmonary embolism in patients with COVID-19 and its treatment based on low-molecular-weight heparin

**DOI:** 10.1590/1516-3180.2020.0221.R2.22072020

**Published:** 2020-08-14

**Authors:** Amin Hosseini, Fatemeh Bahramnezhad

**Affiliations:** I BSc. Master’s Student in Medical-Surgical Nursing Department, School of Nursing and Midwifery, Tehran University of Medical Sciences, Tehran, Iran.; II PhD. Assistant Professor, Nursing and Midwifery Care Research Center, School of Nursing and Midwifery, Tehran University of Medical Sciences, Tehran, Iran. Affiliated member, Spiritual Health Group, Research Center of Quran, Hadith and Medicine, Tehran University of Medical Sciences, Tehran, Iran.

Dear Editor,

In late 2019, a large number of pneumonia patients were diagnosed as having a disease of unknown cause in Wuhan, China. Following this outbreak of human symptoms and infections, a novel coronavirus was identified as the pathogen. In February 2020, the World Health Organization (WHO) named this novel coronavirus COVID-19.^1^ In studies on patients, fever, coughing, myalgia or fatigue were identified as common symptoms, and production of sputum, headache, hemoptysis and diarrhea as less common symptoms. Dyspnea was also reported in about half of the patients. Patients’ blood tests showed normal or low white blood cell counts (25%) and lymphopenia (65%). Highlights of radiological images in patients with severe coronavirus pneumonia also included a ground-glass appearance and lung consolidation that could affect both lungs. Gradually, in a general classification, patients were placed into four categories: mild, moderate, severe and critical, based on the severity of symptoms.^1^^,^^2^

However, one of the clinical findings that has been reported to be relatively higher than expected, in patients with COVID-19 in intensive care units, is pulmonary embolism. Studies conducted around the world have now shown a link between COVID-19 and occurrences of pulmonary embolism and its prevalence.^3^ In a study in France, this clinical finding was estimated to be twice as high in patients with COVID-19 as in those with influenza. That study also reported that out of the 107 patients admitted to the intensive care unit (ICU), 22 (20.6%) patients had pulmonary embolism.^4^ Another study in France reported a rate of 30%.^5^ Zotzmann showed that the laboratory D-dimer levels were elevated in a large number of patients.^6^ Cellina et al. also highlighted the importance of identifying and managing pulmonary embolism in patients with COVID-19.^7^ In a case study conducted by Danzi et al., the possibility of a link between severe infections in these patients and pulmonary embolism was noted.^8^ The results from their study were in line with what had been reported by Grillet et al.^3^ regarding this relationship. The prevalence of pulmonary embolism among hospitalized COVID-19 patients in the latter study was 23%.^3^

Pulmonary embolism is defined as an obstruction in the pulmonary vasculature caused by air, fat, tumor growth or, the most common cause, thrombosis. This condition is known to be life-threatening, and is a cause of death among patients admitted to intensive care units. The main risk factor for this condition is vascular thromboembolism in the lower extremities, which can eventually lead to pulmonary embolism, followed by mechanical and chemical events. Obstruction of the pulmonary vasculature can increase vascular resistance and pressure, and right-sided heart failure. If the pulmonary vascular pressure increases, chemicals are released and this ultimately causes ventilation-perfusion mismatch.^9^

In COVID-19 infection, endothelial cell dysfunction develops, leading to increased thrombin production and cessation of fibrinolysis. This indicates an increase in coagulation status (one of the main risk factors for vascular thromboembolism) in infected patients. In addition, in COVID-19 infection, hypoxia (especially the acute form) can facilitate thrombosis not only through increasing blood viscosity but also through activation of a hypoxia-inducible transcription factor-dependent signaling pathway. This results in obstruction and formation of microthromboses in patients’ small pulmonary arteries, in critical forms of COVID-19.^10^

Oxygen exchange in the lungs can also be disrupted through a reaction between the coronavirus spike (S) protein and the angiotensin-converting enzyme 2 (ACE 2) receptor in the lungs, thereby increasing ACE 2 expression. Studies have shown that ACE 2 has a greater tendency to bind to coronaviruses. This can eventually destroy the alveoli and reduce oxygen exchange.^11^ Injury to alveolar cells can, in turn, cause a series of systemic reactions and even death.^12^

Respiratory and hemodynamic support, along with anticoagulant therapy, are the mainstay of pulmonary embolism treatment. The American College of Chest Physicians guidelines now emphasize treatment of acute pulmonary embolism as soon as possible, using parenteral anticoagulants. These can include unfractionated heparin, fondaparinux or subcutaneous low-molecular-weight heparin (LMWH).^9^ LMWH is preferred in these patients because of its cost, availability and authorization for use.^13^ Use of heparin as an experimental anticoagulant in patients with COVID-19 and high D-dimer levels has reportedly been associated with lower mortality.^6^ Experts have recently emphasized the desirability of management of coagulopathy in all COVID-19 patients using prophylactic doses of LMWH.^14^

Poissy et al. pointed out that heparin can have positive effects in patients with COVID-19, but noted that uncertainties remained regarding the heparin dose that would be most effective, and regarding monitoring of complications.^4^ Treatment with heparin reduces inflammatory biomarkers and may, therefore, be effective in reducing the inflammatory status of COVID-19. Heparin is essential for limiting fibrin deposition and microthrombus formation, and for treating systemic prothrombotic complications in patients. However, it is ineffective for clearing fibrin clusters in the alveolar space.^15^

It seems that further studies are needed in order to justify prescription of LMWH as prophylaxis for prevention of pulmonary embolism in patients with COVID-19.
